# Reconciling Coronary Artery Calcification in the Lipid-Lowering Era

**DOI:** 10.1016/j.jacadv.2025.102506

**Published:** 2026-01-28

**Authors:** Shaun Khanna, Nitesh Nerlekar, Aditya Bhat

**Affiliations:** a The George Institute for Global Health, UNSW Sydney, New South Wales, Australia; b Victorian Heart Institute, Victorian Heart Hospital, Clayton, VIC, Australia; c Department of Cardiology, Westmead Hospital, New South Wales, Australia

**Keywords:** coronary artery calcium, lipid-lowering therapy

In modern cardiovascular prevention, achieving a reduction in low-density lipoprotein cholesterol (LDL-C) levels has become central to risk mitigation. High-intensity statins and, more recently, proprotein convertase subtilisin/kexin type 9 (PCSK9) inhibitors enable unprecedented lipid lowering, translating into substantial reductions in atherosclerotic cardiovascular events.^1^^,^^2^ Yet, a persistent paradox challenges clinicians; despite clear reductions in events, coronary artery calcium (CAC) often increases under these therapies.^3^ This mechanism appears counterintuitive; how can interventions that reduce plaque burden and stabilize disease lead to rising CAC scores, which is traditionally a marker of overall risk. Understanding this discrepancy requires re-examining what CAC truly represents in the context of plaque biology, therapy-induced remodeling, and modern imaging.

## Defining the coronary calcium paradox

CAC scoring by noncontrast computed tomography (CT) has long been among the most powerful predictors of future atherosclerotic events.^4^ Individuals with high CAC scores have substantially elevated risk compared with those with zero calcium. However, longitudinal data reveal that lipid-lowering therapy, particularly statins, can lead to apparent CAC progression despite reductions in myocardial infarction and mortality.^5^ Serial imaging from large cohorts shows that patients on statins exhibit greater increases in CAC over time than untreated counterparts, even as plaque volume decreases and clinical outcomes improve.^5^

The central insight is that CAC is not a direct measure of atheroma volume but illustrates a reflection of plaque composition and chronic remodeling. In untreated disease, calcification signifies cumulative injury and necrosis; in treated disease, it may reflect healing and stabilization. Recognizing this dual nature is key to reconciling this paradox.

## Mechanisms and remodeling

The most widely accepted explanation is that intensive lipid lowering transforms unstable, lipid-rich plaques into more stable, calcified lesions. Histopathologic and intravascular imaging studies have shown that statins promote resolution of lipid pools, suppression of inflammation, and deposition of dense calcium that caps necrotic cores.^6^ This process produces fewer rupture-prone plaques, even though the overall calcium burden measured by CT increases. The “healing hypothesis” posits that what we detect radiographically as new calcium represents the end stage of plaque maturation rather than new atherogenesis.

As lipid and necrotic debris are cleared, microcalcifications can merge into larger macrocalcifications visible on CT. This coalescence increases the Agatston score but reduces mechanical stress on the fibrous cap. In other words, more calcium can mean less danger. Statin-induced increases in calcium density, rather than volume, are particularly associated with greater stability and lower event rates.^7^ PCSK9 inhibitors appear to amplify these effects, further thickening fibrous caps and reducing lipid arcs without necessarily decreasing total CAC.^8^

**Figure 1:**
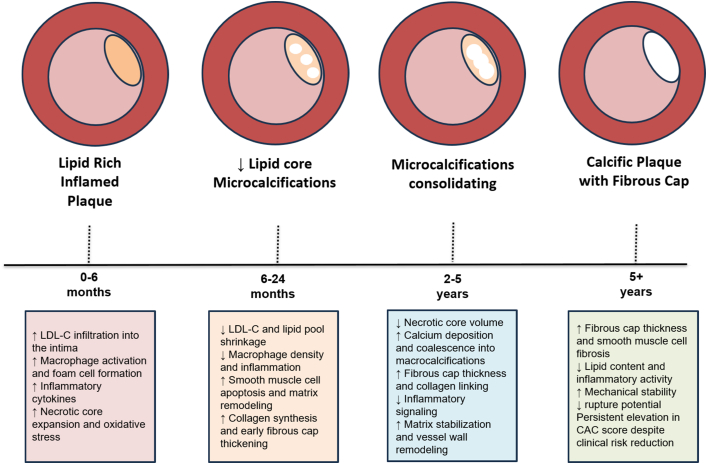
**Lipid-Lowering Therapy and Coronary Artery Calcification** Conceptual transition of atherosclerotic plaque under intensive lipid-lowering, from an inflamed, lipid-rich state to a more fibrotic, calcified, and mechanically stable lesion over time, despite rising calcium scores. CAC = coronary artery calcium; LDL-C = low-density lipoprotein cholesterol.

Temporal dynamics also play a role. Lipid and inflammatory components regress rapidly, whereas calcification progresses more slowly, often lagging by months to years.^9^ Thus, early increases in CAC after therapy initiation likely represent a transient phase in the remodeling trajectory. Moreover, CT imaging artifacts, such as blooming and partial-volume effects, can exaggerate apparent progression when plaque composition changes or density increases.^9^ These technical limitations contribute to the illusion of worsening despite biological improvement. See Figure 1.

## Evidence from the large-scale lipid-lowering studies

Serial intravascular ultrasound and optical coherence tomography studies have demonstrated that intensive statin therapy reduces total atheroma volume, increases calcium density, and thickens fibrous caps.^10^ With the addition of PCSK9 inhibitors, these morphological changes are accentuated: total and percent atheroma volumes decline further, necrotic core and lipid arcs shrink, and the fibrous cap becomes more robust. Notably, these compositional shifts occur alongside increases in calcification indices, indicating that calcium accumulation and plaque stabilization can coexist.

Large trials such as FOURIER (Further Cardiovascular Outcomes Research With PCSK9 Inhibition in Subjects With Elevated Risk) and ODYSSEY OUTCOMES (Alirocumab and Cardiovascular Outcomes after Acute Coronary Syndrome) have shown that LDL-C reductions achieved through PCSK9 inhibition produce incremental event reductions beyond statin therapy alone, despite no evidence that calcium regression accompanies these gains. Taken together, these findings indicate that CAC progression under lipid-lowering therapy does not equate to therapeutic failure; instead, it may represent structural healing.

## Interpreting rising CAC in clinical practice

In clinical care, interpreting CAC progression requires restraint and contextualization. Short-term increases in CAC following initiation of statins or PCSK9 inhibitors should not prompt alarm or escalation of therapy if LDL-C targets are achieved and the patient remains asymptomatic. Rather, these changes should be viewed as evidence of lesion stabilization. The key is to integrate CAC data with complementary imaging and clinical information. Coronary CT angiography, for example, can differentiate low-attenuation, lipid-rich plaque from densely calcified, fibrotic plaque, providing a more precise assessment of risk. A rise in CAC accompanied by a reduction in noncalcified plaque volume, improved remodeling index, or diminished pericoronary inflammation is likely benign, or even favorable. Clinicians should emphasize that the goal of therapy is reduction in events and mortality, not necessarily imaging regression.

## Management implications

Recent analyses from the CAC Consortium, highlighted by the American College of Cardiology, reaffirm that the Agatston CAC score continues to predict cardiovascular events among statin users, though its prognostic strength is attenuated compared with statin-naïve individuals. Importantly, it is highlighted that CAC volume, rather than density, retains discriminatory value in such populations. When available, CAC volume (mm^3^) can be tracked longitudinally, as these are less affected by statin-induced plaque densification and better reflect total disease burden. Furthermore, very high CAC scores (ie, >400 or >1,000) remain markers of extensive atherosclerosis warranting aggressive additional preventive therapy in patients on established lipid-lowering therapy.

## Implications for research

The paradox also has implications for how we design and interpret endpoints in clinical research. Reliance on CAC progression as a surrogate for treatment efficacy is increasingly weak. Future lipid-lowering trials should incorporate measures of plaque vulnerability, such as low-attenuation plaque burden, positive remodeling, fibrous cap thickness, and inflammation, rather than total calcium alone. The integration of radiomics and artificial intelligence could refine our ability to distinguish harmful calcification from protective remodeling.

There also remains a need to understand interindividual variability, as not all patients exhibit the same calcific response to therapy. Genetic predisposition, metabolic background, and inflammatory pathways may influence whether plaques predominantly calcify or regress in lipid content. Personalized imaging and lipid-lowering strategies may ultimately emerge from this knowledge, ensuring that calcium progression is interpreted within the context of each patient’s biological phenotype.

## Conclusions

The coronary calcium paradox challenges simplistic interpretations of imaging biomarkers in cardiovascular medicine. Rather than representing treatment failure, rising CAC under statin or PCSK9 inhibitor therapy likely reflects a process of plaque stabilization and healing, a biological success disguised as radiographic progression. Clinicians should interpret these findings through a pathophysiologic lens, integrating plaque composition, clinical outcomes, and therapeutic context rather than relying on calcium scores alone.

## Funding support and author disclosures

The authors have reported that they have no relationships relevant to the contents of this paper to disclose.
